# Unveiling the immunometabolic landscape of colorectal cancer through PANoptosis-related gene expression

**DOI:** 10.3389/fimmu.2025.1615022

**Published:** 2026-01-12

**Authors:** Xiaoyu He, Wenhao Wang, Li Li, Yiru Yin, Shunbin Ding

**Affiliations:** 1 Department of Gastroenterology, People’s Hospital of Deyang City, Deyang, Sichuan, China; 2 Department of Obstetrics and Gynecology, The Second Clinical Medical College, Shanxi Medical University, Taiyuan, Shanxi, China; 3 Department of Geriatrics, The Second Hospital of Shanxi Medical University, Taiyuan, Shanxi, China; 4 Key Laboratory of Cellular Physiology, Ministry of Education, Department of Physiology, Shanxi Medical University, Taiyuan, China

**Keywords:** CDKN2A, colorectal cancer (CRC), immune microenvironment, PANoptosis, single-cell RNA sequencing

## Abstract

**Background:**

Colorectal cancer (CRC) development and progression are linked to genetic factors, environmental influences, and dysregulated signaling pathways.

**Methods:**

The differentially expressed pan-apoptotic genes (CPAN_DEGs) between CRC and normal colon tissues were screened from bulk RNA-sequencing (RNA-Seq) datasets. The putative biological functions of these CPAN_DEGs were explored through functional enrichment analysis and the protein-protein interaction (PI) network. Unsupervised clustering was used to stratify patients on the basis of CPAN_DEGs, and a prognostic model was constructed using LASSO dimensionality reduction. Based on the CPAN-index score, the patients were divided into the high-risk and low-risk groups, and the survival rates and immunophenotypes were compared. The predictive performance of the CPAN-index model was confirmed in an external validation set. The expression patterns of PANoptosis genes across different cell types in CRC samples, and the distribution of CPAN-index-positive cells within each subpopulation were analyzed using single cell RNA-Seq (scRNA-Seq) datasets. The expression of CDKN2A was confirmed in CRC cell lines, and its functional role was evaluated by gene knockdown.

**Results:**

The expression levels of PANoptosis-related genes showed significant heterogeneity across CRC samples, and the highest percentage (87.4%) was that of apoptosis-related genes. The differentially expressed genes (DEGs) between the CRC and normal tissue samples were significantly enriched in pathways related to metabolism and immune regulation. The CPAN-index constructed using 11 CPAN_DEGs effectively distinguished CRC patients in to the high-risk and low-risk groups, and the high-risk group showed an “invasion-metabolism-immunosuppressive” phenotype, along with immune tolerance and non-classical immune escape. The CPAN-index gene CDKN2A was upregulated in the CRC cell lines, and knocking down the CDKN2A gene inhibited their proliferation and promoted apoptosis *in vitro*. ScRNA-Seq data revealed a higher proportion of CPAN-index-positive immune cells, and a lower proportion of tumor cells positive for CPAN-index, thus underscoring its critical role in the tumor immune microenvironment.

**Conclusions:**

CDKN2A-mediated PANoptosis signaling network drives CRC progression by reshaping the immune microenvironment and metabolic reprogramming. The CPAN-index provides a new tool for accurate risk stratification of CRC patients, and suggests potential therapeutic strategies targeting the immunometabolism-death interaction network.

## 1 Introduction

Colorectal cancer (CRC) was the third most prevalent malignancy worldwide in 2020, with 1,931,590 newly diagnosed cases that accounted for 10% of all cancers. In addition, the mortality rate associated with CRC was 9.4%, with approximately 935,173 recorded deaths (1, 2). Given its high incidence and mortality, as well as the rise in incidence among individuals previously considered to be at low risk (3), CRC poses a significant economic burden. Smoking, drinking and weight gain are among the risk factors of CRC (4), whereas a healthy diet can reduce the risk associated with colon cancer (5) (6) (7). Mechanistically, CRC initiation and progression involve aberrant cellular proliferation and differentiation, along with increased resistance to apoptosis (8, 9). At the molecular level, pathogenesis of CRC is associated with the overactivation of phosphoinositol 3-kinase (PI3K)/AKT and transforming growth factor-β (TGF-β) signaling pathways due to multiple oncogenic mutations (10). The clinical manifestation of CRC, including fatigue, anemia, and weight loss have limited predictive power for CRC in older patients. As CRC screening programs become more widely available, many cases are detected prior to the appearance of symptoms (11). For symptomatic patients, the preferred diagnostic method is colonoscopy, although other endoscopic techniques may be selected. The primary radical treatment for patients with non-metastatic CRC is surgery (6, 12). In recent years, immunotherapy has gradually been incorporated for the treatment of advanced cases in combination with radiotherapy (13). Nevertheless, it is essential to elucidate the molecular mechanisms underlying the pathogenesis and development of CRC in order to devise more effective and personalized therapeutic strategies.

Programmed cell death, including pyroptosis, apoptosis, and necrosis, is the central mechanism of most anti-cancer therapies and the immune defense against tumor cells (11, 14, 15). As the three cell death modes overlap each other, Malireddi et al. proposed the concept of PANoptosis, which is stimulated by particular triggers and controlled by the PANoptosome complex (16, 17). Upstream receptors such as ZBP1, RIPK1, and AIM2 detect particular stimuli and initiate the formation of the PANoptosome (18, 19). Recent studies indicate that PANoptosis is significantly linked to the onset and progression of infectious diseases and cancer (20), thereby warranting a thorough exploration of the mechanisms underlying PANoptosis to formulate effective treatment strategies (21). In this study, we analyzed the role of PANoptosis-related genes in the prognosis and immunomodulation of CRC.

## 2 Methods

### 2.1 Data retrieval

**Figure 1:**
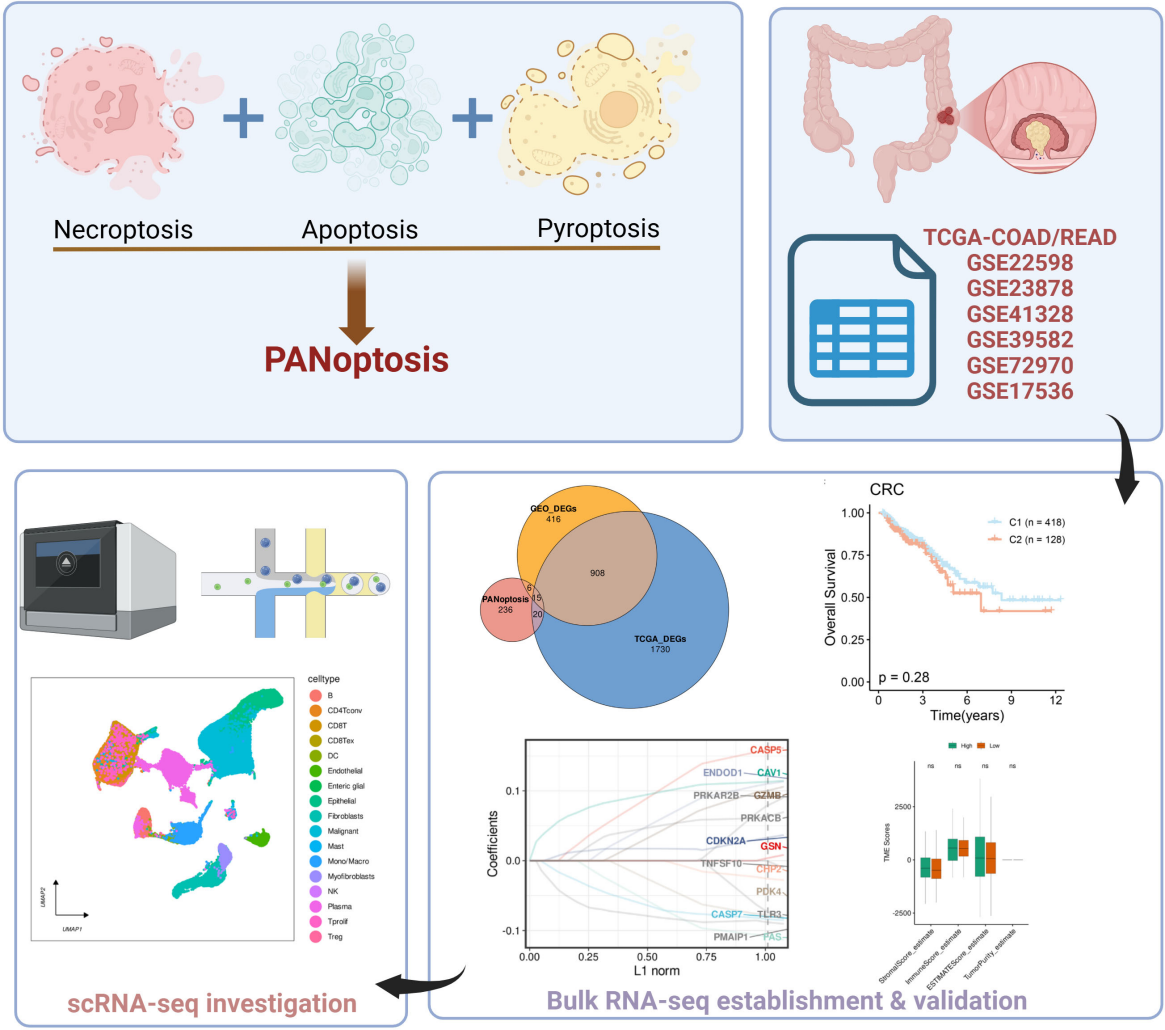
The flow chart of this study.

The Bulk of colorectal adenocarcinoma (COAD) and colorectal adenocarcinoma (READ) were downloaded from NHGRI databases RNA-seq and batch removal via the “sva” packet combat function are finally merged into the CRC data set. We downloaded Bulk RNA-seq (GSE41328, GSE22598, GSE41328, GSE22598) from NCBI and NIH for colorectal cancer. GSE23878 and based on the “IOBR” R package (https://github.com/IOBR/IOBR) remove_batcheffect function merge is used to calculate carcinoma and genetic variations. In addition, Bulk RNA-seq (GSE39582, GSE72970, GSE17536) obtained from this data source contained prognostic information to assess the efficacy of the prognostic model. Single-cell RNA-Seq (scRNA-Seq) data related to CRC was obtained from the TISCH database (http://tisch.comp-genomics.org/home/), and included the GSE146771, EMTAB8107, and GSE166555 datasets (22). The missing samples were eliminated. In case of multiple rows for a gene in the expression matrix, the average was calculated. All data sources used in this study are publicly available and can be accessed without additional ethical approval. Pan-apoptotic genes were obtained from a supplementary dataset from literature (23). The research flow is shown in **Figure 1**.

### 2.2 Biological and pathway analysis

Based on the “limma” R package, we identified cancer and paratancerecancer differential genes (|logFC|> 1&p.Val < 0.05) in the combined CRC dataset and cancer and paratancerecancer differential genes (|logFC|> 1&p. Val < 0.05) in the GEO dataset, and performed heat map visualization. Based on the intersection of differential genes from two datasets, the common differential expression panapoptosis genes (CPAN_DEGs) were obtained, and then the protein interaction network (PPI) of these genes was constructed (24, 25).

### 2.3 Unsupervised clustering and correlation analysis

The “ConsensusClusterPlus” R package was used to conduct an unsupervised clustering analysis on the CPAN_DEGs. Proportional fuzzy clustering (PAC) and cumulative distribution function (CDF) curves (26) were used to find the most suitable group (k=2 in this analysis), and the prognosis of the two clusters was compared. The DEGs between the two clusters were identified using the “limma” R package (|logFC|>1 & p. Val < 0.05). CIBERSORT and ESTIMATE algorithms were used to analyze the immune microenvironment and immune checkpoint expression in both clusters.

### 2.4 Construction and verification of prognostic model

The prognostic value of the DEGs was evaluated in the CRC cohort by univariate Cox analysis (27). Through a systematic alteration of the regularization parameter log lambda, we observe the convergence pattern of Binomial Deviance to identify the ideal lambda value, which reflects the best equilibrium between the complexity of the model and its predictive efficiency. Based on this optimization parameter, the coefficient path plot of gene coefficient variation with L1 norm was plotted, and 15 candidate genes with significant prognostic value were incorporated in the multivariate Cox proportional risk model. The coefficients were calculated by the maximum partial likelihood estimation method to quantify their independent predictive power for survival outcomes. Based on the variable compression characteristics of LASSO regression and the results of survival analysis in the Cox model, the “CPAN-index” prognostic model was established. The coefficient distribution of model genes was determined by a lollipop diagram. The predictive performance of the model was tested on the GSE39582, GSE17536, and GSE72970 datasets by receiver operating characteristic (ROC) curve analysis, and the area under the curve was calculated. In addition, the association between the individual risk scores and survival duration was determined for each dataset.

### 2.5 Analysis of immune characteristics

The immune profile of the CRC dataset was analyzed using the CIBERSORT algorithm (28). To investigate the variations among CRC samples categorized as h-risk and l-risk, we initially conducted an analysis of the merged data set for CRC. Through the data set to submit to the TIDE’s, we get the samples of each CRC score or express quantity on several key indicators, The dysfunction score, CD274 expression, IFNG, TIDE score, Merck18, and CD8 were included and visualized to observe the immunosuppression characteristics difference. Gene-set enrichment analysis (GSEA) was performed using the “GseaVis” package (29). The initial key pathways were delineated to illustrate the enrichment of genes unique to both the high-risk and low-risk categories. Furthermore, the expression of immune checkpoint genes in both risk groups were also analyzed.

### 2.6 Single cell analysis

Three CRC scRNA-seq datasets were integrated, followed by batch removal through the “harmony” R package, and automatic annotation using “SingleR” data provided by TISCH. The single cell data was reduced to two dimensions using the Uniform Manifold Approximation and Projection (UMAP) algorithm, and the cell distribution of Seurat clusters, cell type and each data set was visualized. The genes with significant variations in expression across various cell types were screened using the FindMarkers function in Seurat. The upregulated and downregulated genes within each primary cell type were visualized using a Volcano plot. The AddModuleScore function in Seurat was used to score the predefined CPAN-index gene modules in the single-cell dataset in order to quantify the expression of CPAN-index genes in each cell. Furthermore, the proportion of CPAN-index positive cells in the different populations were calculated.

### 2.7 Cell culture and transfection

The normal colon epithelial cell line HIEC-6, and the CRC lines LoVo, HT-29, HCT116 and SW480 were obtained from Cell Bank of the Chinese Academy of Sciences. The HIEC-6 cells were cultured in OGM BulletKit medium (Lonza, Switzerland). HCT116 and SW480 cell lines were transfected with siRNA constructs specific for the CDKN2A gene, and a negative control (NC) construct (Sangon, China). Briefly, once the cells were 80% confluent in the 6-well plate, the lipo3000 transfection reagent and siRNA are diluted using Thermo’s (USA) Opti-MEM reduced serum medium. After 5 minutes, mix the two solutions and leave for another 20 minutes. The prepared transfer solution was introduced into the six-well plate, and the culture medium was refreshed 5 hours after transfection.

### 2.8 Total RNA extraction and RT-qPCR

The cells were harvested and lysed with 950 μL Trizol reagent (Takara, Japan) in the presence of RNase inhibitors. After incubating for 5 minutes, 150 μL chloroform (Sinopharm Holdings, China) was added to each tube, and the samples were mixed by vortex oscillation and centrifuged for 5 minutes. The supernatant was collected, and isopropyl alcohol (SINOPHARM, China) was added to precipitate the RNA. The samples were centrifuged for 5 minutes, and the RNA pellets were washed with 1 mL 75% or anhydrous ethanol, and air dried. The RNA concentration, and the level of DNA and protein contamination were measured. Following removal of genomic DNA, the RNA samples were reverse transcribed to cDNA using the PrimeScript RT kit (TaKaRa, Japan) as per the manufacturer’s guidelines. RT-qPCR was performed using the SYBR GreenER Supermix (TaKaRa, Japan) on the Roche480 PCR system (Roche, Switzerland).

### 2.9 Cell counting kit-8 assay

One day post-transfection, the cells were seeded into 96-well plates at the density of 4000 cells per well, and three replicate wells were set up for each group. At pre-determined time points, the CCK8 solution (KeyGEN, China) diluted in complete medium to a final volume of 100 µl was added to each well. The cells were incubated for 1.5 hours, and the absorbance of each well at 450nm was measured using a microplate reader.

### 2.10 Transwell migration assay

After transfection, we digested cells to harvest them and resuspended in serum-free medium. To ensure result accuracy, we seeded cells at 40,000 per well, with 250 μl suspension in each chamber, and added 550 μl complete culture medium to each 24 well plate well. Then we put chambers into plates and incubated for 24 hours. After that, we removed chamber fluid, wiped off non invaded cells, fixed cells with paraformaldehyde for 30 minutes, washed chambers twice with PBS, stained cells with 0.1% crystal violet for 20 minutes, washed again, and let them air dry. Finally, we took chamber images under a microscope for further analysis.

### 2.11 Flow cytometry

We employed flow cytometry to measure apoptosis. First, the cells were digested using EDTA free trypsin (Beyotime, China) and then centrifuged at 2,000 rpm to collect the cell pellets. Next, each group of cells was resuspended in 500 μL of Binding Buffer (Biosharp, China) and transferred into a flow tube. Following the manufacturer’s instructions, each group was supplemented with 5 μL of propidium iodide (PI, Biosharp, China) and 5 μL of fluorescein 5-isothiocyanate (FITC, Biosharp, China). After staining the cells in a 37°C water bath for 15 minutes, they were analyzed by flow cytometry. This entire process was repeated three times for each group.

### 2.12 Wound healing assay

HCT116 and SW480 cells were seeded in a 6-well plate (5×10^5^ cells per well). After 24 h incubation, a vertical scratch lines was generated in each well. After washing with PBS, serum-free medium was added for cell culture. The images were taken at 0 h and 48 h. The experiment was repeated thrice.

### 2.13 Statistical analysis

All data in this study were based on at least 3 biological replicates, and all statistical studies used R software (version 4.4.1). The t test was used for comparison between groups, and continuous data were expressed as mean ± standard deviation (mean ± SD). P values < 0.05 were deemed statistically significant, and all P values were tested in a two-sided manner.

## 3 Results

### 3.1 Identification and functional annotation of differentially expressed PANoptosis genes

**Figure 2:**
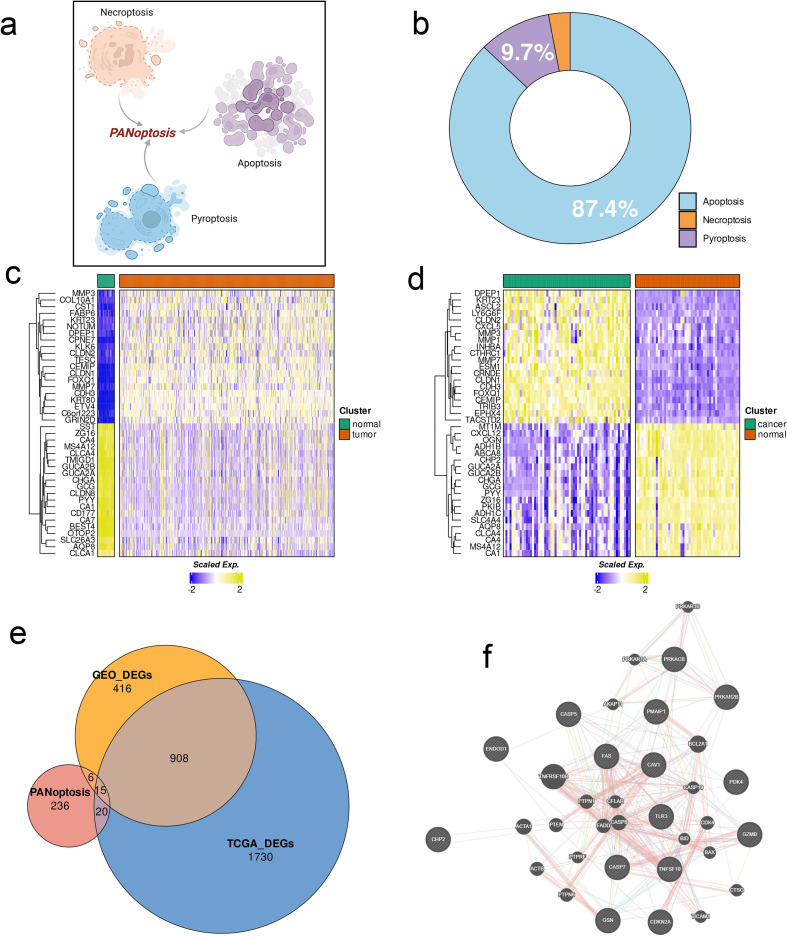
Identification of PANoptosis-related DEGs (CPAN_DEGs) in CRC. **(A)** PANoptosis concept map (created by BioRender). **(B)** The proportion of apoptosis, necrosis and pyroptosis-related genes in the PANoptosis gene list. **(C, D)** Heat maps of the top 20 up-regulated and down-regulated genes between CRC and normal tissue in TCGA and combined GEO databases. **(E)** Venn map showing the overlap of DEGs and PANoptosis gene set. **(F)** PPI network of differentially expressed PANoptosis genes (CPAN_DEGs).

**Figure 3:**
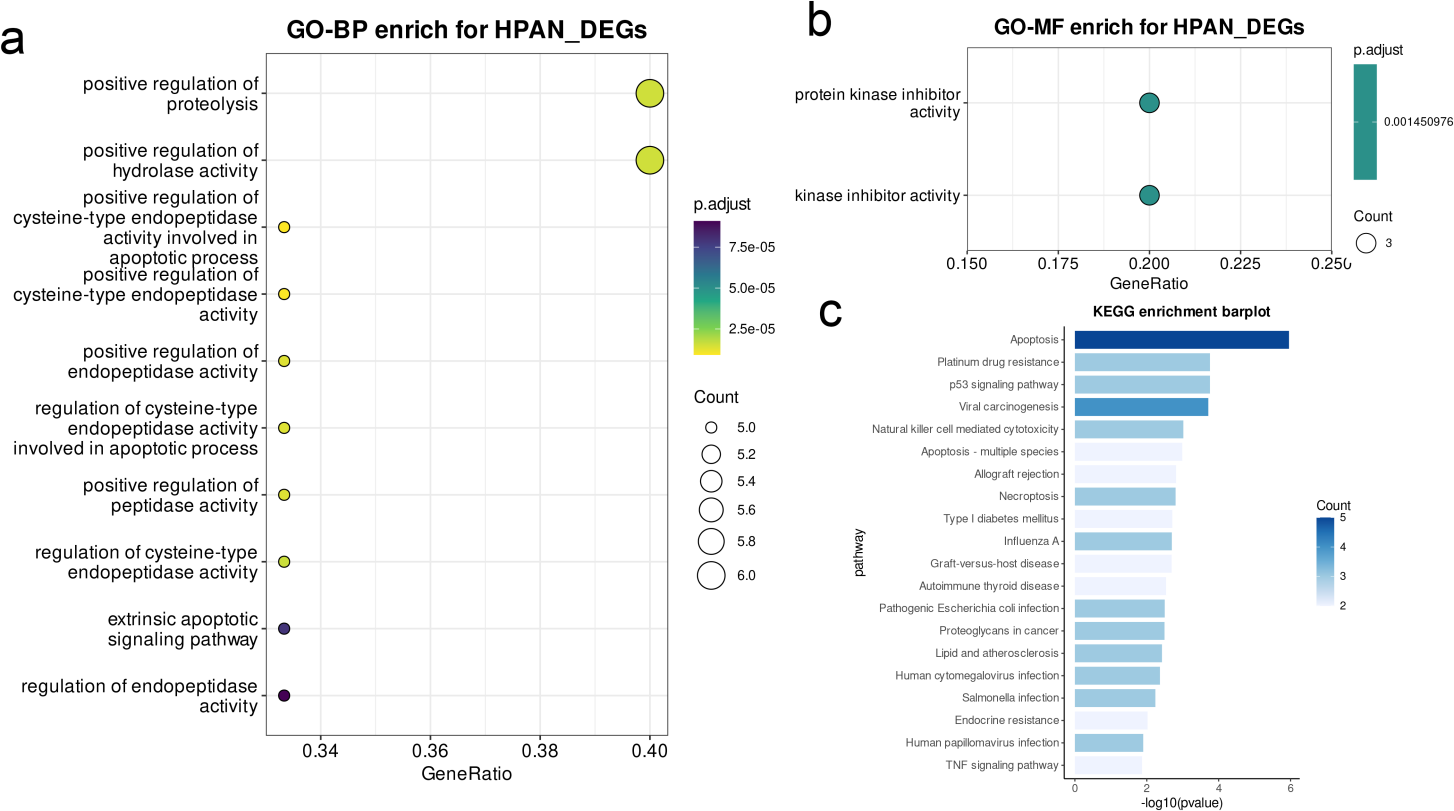
GO/KEGG/GSEA enrichment analysis of CPAN_DEGs. **(A, B)** Significantly enriched GO-BP **(A)** and GO-MF **(B)** terms in CPAN_DEGs. **(C)** KEGG pathways significantly associated with CPAN_DEGs.

The concept of PANoptosis has been outlined in **Figure 2a**. The apoptosis-related genes comprised 87.4% of the PANoptosis gene list, whereas 9.7% and 2.9% of the genes were respectively associated with pyroptosis and necroptosis (**Figure 2b**). The top 20 DEGs between the CRC and normal colon tissues in the CRC and combined GSE databases are shown in **Figures 2c–d**. The overlap of DEGs in the CRC and GSE datasets with the PANoptosis gene list was determined by plotting Venn diagrams, and a total of 908 DEGs were common to both datasets, of which 15 genes were part of the PANoptosis list (**Figure 2e**). The protein-protein network (PPI) of these genes was constructed, and as shown **Figure 2f**, TNFRSF10B, FAS, CAV1, and PTPN1 were among the core genes of the network. The CPAN_DEGs were functionally annotated on the basis of Gene Ontology (GO) terms and KEGG pathways. The significantly enriched terms related to biological processes (GO-BP) included “enhancing proteolysis,” “promoting hydrolase activity,” and “facilitating cysteine-type endopeptidase activity”, and in particular “positively regulating proteolysis” and “positively regulating hydrolase activity” (p.adj < 0.0001, **Figure 3a**). Furthermore, “protein kinase inhibitor activity” and “kinase inhibitor activity” were among the molecular function terms (GO-MF) showing significant enrichment (p.adj < 0.01; **Figure 3b**). Analysis of the KEGG database revealed that multiple pathways related to apoptosis, immune response and viral infection were significantly enriched in the CPAN_DEGs, and the “apoptosis” pathway showed the highest enrichment. In addition, the “platinum drug resistance”, “p53 signaling pathway”, “viral carcinogenic effect” and other pathways also showed significant enrichment, while those associated with “human papillomavirus infection” and “TNF signaling pathway” were also enriched to some extent (**Figure 3c**).

### 3.2 Unsupervised clustering and correlation analysis

**Figure 4:**
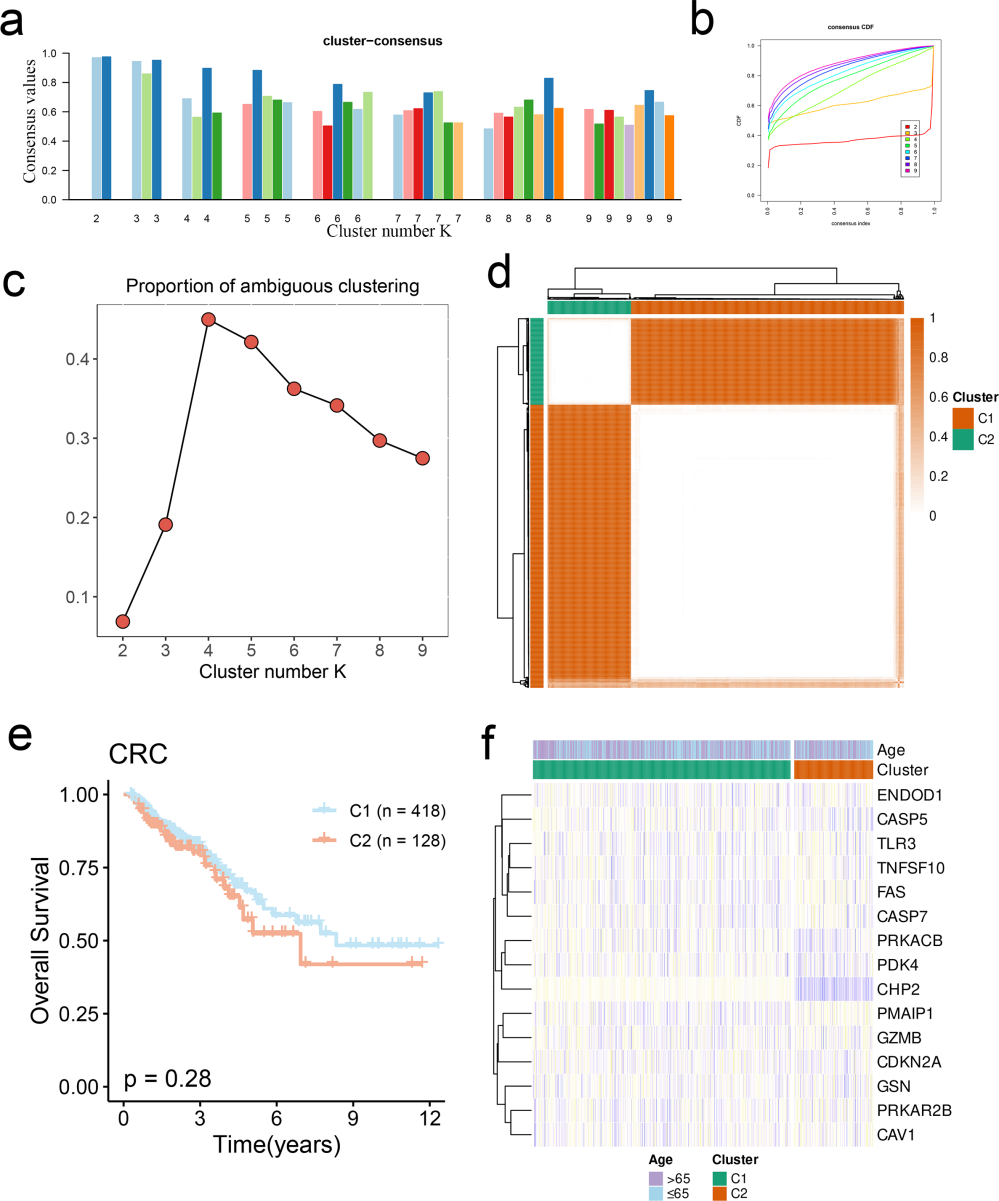
Clustering of CRC samples based on CPAN_DEGs. **(A, B)** Evaluation of mean consistency in clusters and area under CDF curve when k = 2 ~ 10. **(C)** The PAC score indicates the optimal number of subtypes in the CRC dataset. **(D)** The CRC cohort was divided into two CRC subtypes by consensus clustering. **(E)** Kaplan-Meier survival analysis of CPAN_DEGs clusters. **(F)** Heat maps showing the expression of CPAN_DEGs in different CRC clusters.

Unsupervised clustering was performed on the CRC samples based on the CPAN_DEGs. Both intra-cluster consistency (**Figure 4a**) and smoothness under the CDF curve (**Figure 4b**) decreased with the increase in k value. As shown in the PAC diagram in **Figure 4c**, the value is lowest when k=2, indicating that the sample distribution is less affected by random perturbations. Accordingly, the CRC cohort was divided into the C1 cluster (n=418) and the C2 cluster (n=128) (**Figure 4d**). The Kaplan-Meier curves of the two clusters showed no significant difference in survival (p > 0.05, **Figure 4e**). However, there were obvious differences in the expression patterns of CPAN_DEGs between C1 and C2 (**Figure 4f**).

### 3.3 Analysis of immunophenotypes and mutations

**Figure 5:**
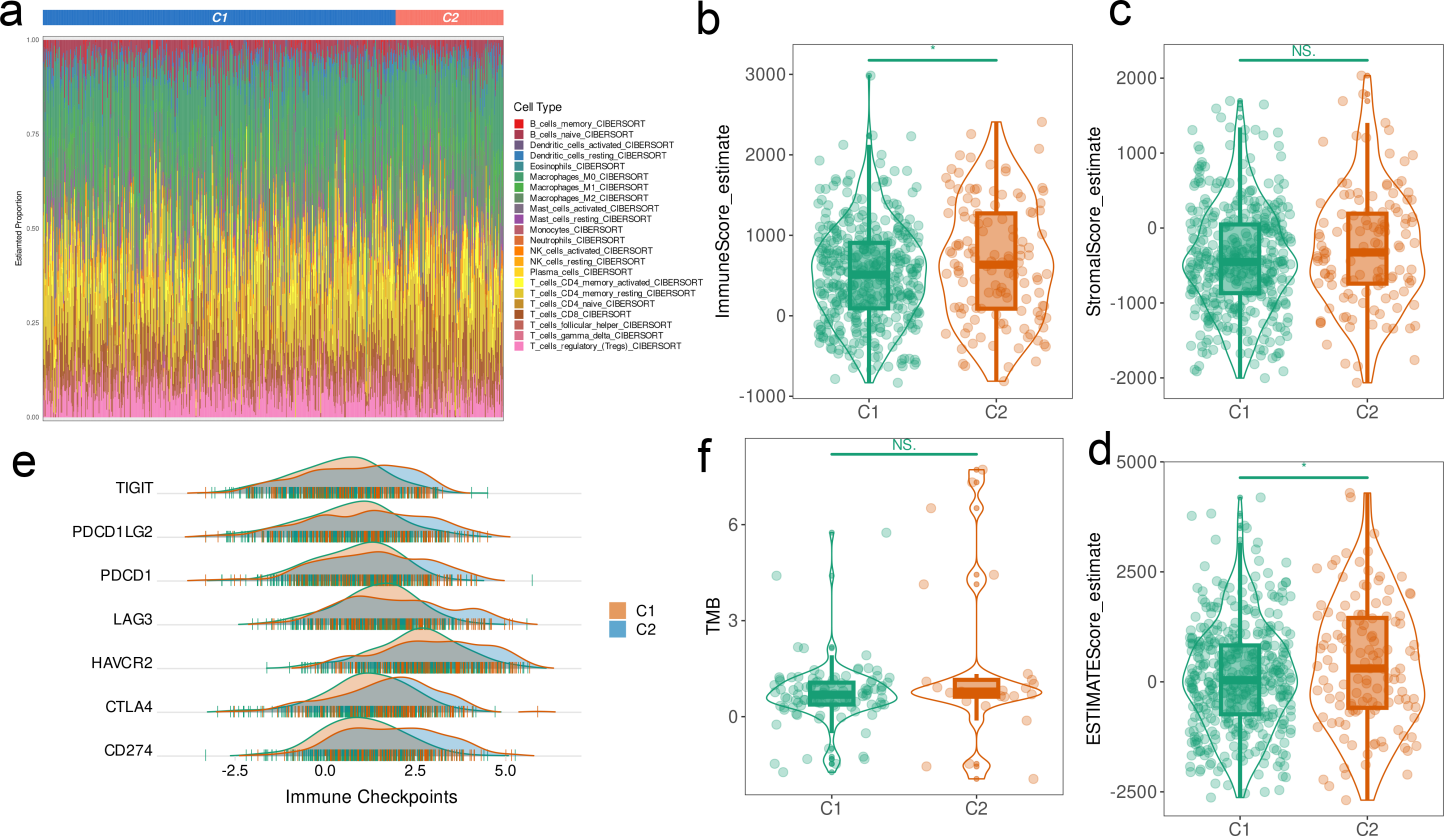
Immunophenotyping of CPAN_DEGs clusters. **(A)** Waterfall map of the distribution of 24 immune cells in CRC clusters. **(B, C)** Immune scores and matrix scores in CRC clusters. **(D)** Microenvironment scores in the CRC clusters. **(E)** Ridge map showing differences in immune checkpoint molecules between the CRC clusters. **(F)** Tumor mutation load in the CRC clusters. (*p < 0.05).

We observed unique immunophenotypes and mutational landscapes across the CRC samples. As shown in **Figure 5a**, the immune score for the C2 cluster was considerably greater than that for the C1 cluster (p < 0.05); however, no significant difference was observed in the matrix score (**Figure 5b, c**). This suggests that the C2 cluster may have stronger immune activity. Furthermore, comparison of the ESTIMATE scores revealed higher microenvironment score in the C2 cluster compared to that of C1 cluster (p < 0.05) (**Figure 5d**), which further supports greater immunoreactivity in the former. However, the expression levels of immune checkpoints such as TIGIT, PDCD1LG2, PDCD1, LAG3, HAVCR2, CTLA4, and CD274 was significantly higher in the C2 cluster than in C1cluster, which may reflect an immunosuppressive state in C2 cluster (**Figure 5e**). The tumor mutation burden (TMB) of the CPAN_DEGs clusters were similar (**Figure 5f**).

### 3.4 Establishment and validation of PANoptosis-related prognostic model

**Figure 6:**
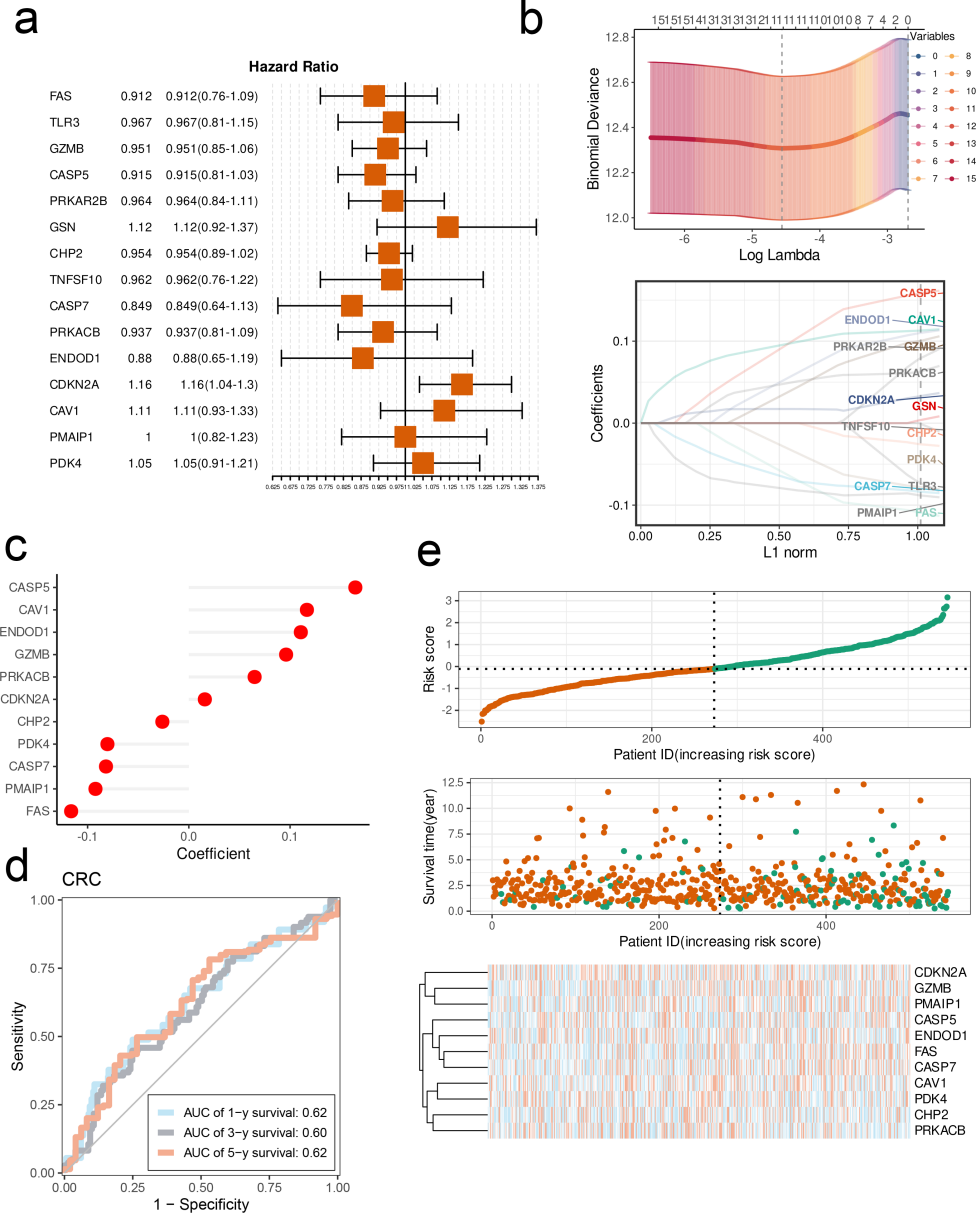
Construction of a PANoptosis risk score model (CPAN-index) for CRC based on CPAN_DEGs. **(A)** Forest maps showing the prognostic value of 15 CPAN_DEGs in the CRC cohort. **(B)** Selection of model CPAN_DEGs by 10-fold cross-validation Lasso regression analysis. **(C)** Lollipop chart showing the coefficients for each CPAN_DEG included in CPAN-index. **(D)** Receiver operator characteristic (ROC) curves of CPAN-index for 1-, 3-, and 5-year survival. **(E)** Heat maps of CAPN-index distribution, survival status, and expression levels of model genes.

**Figure 7:**
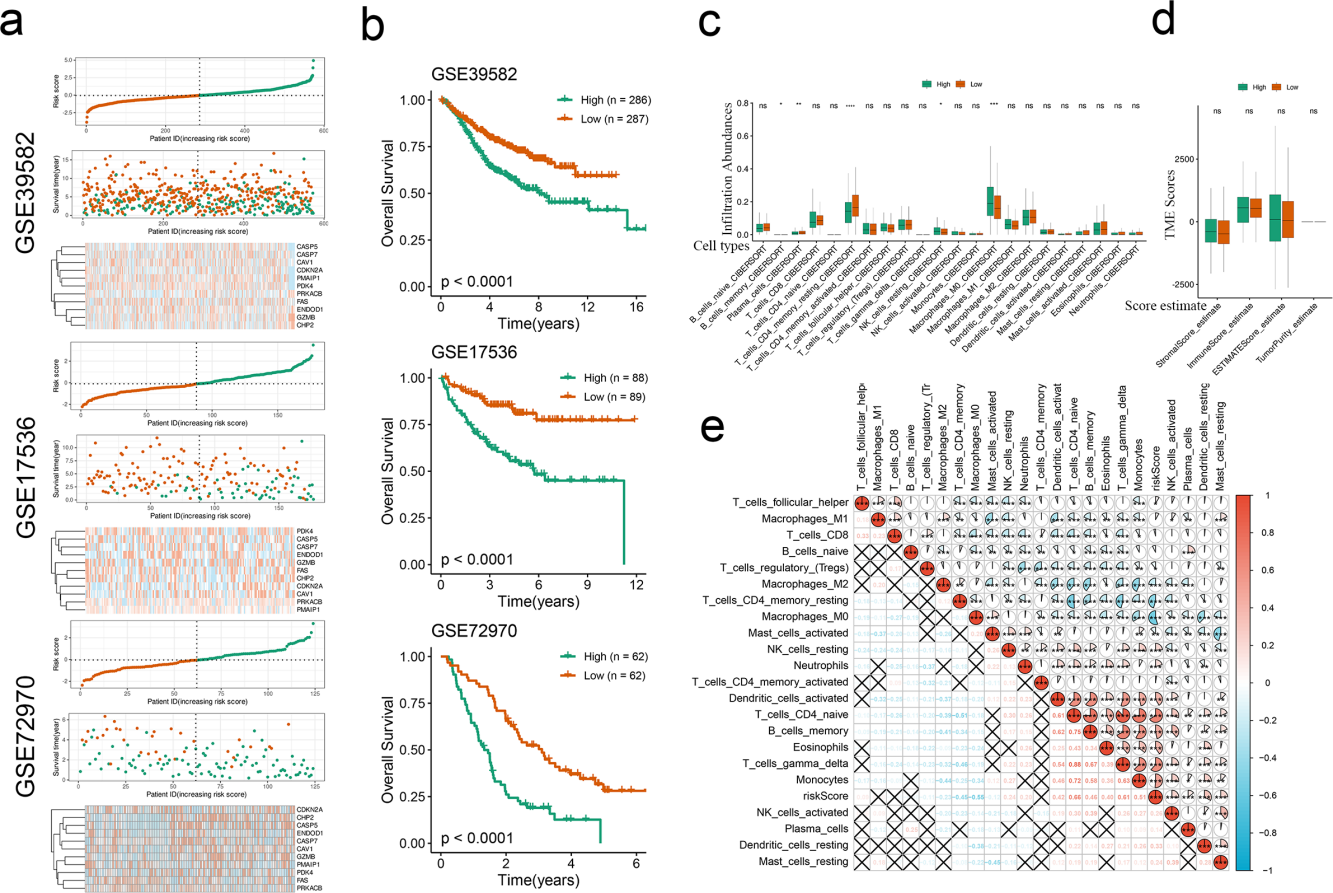
Validation of CPAN-index in multiple CRC cohorts. **(A)** Thermal maps of CPAN-index distribution, survival status, and expression levels of model genes in the three GEO validation datasets. **(B)** Kaplan-Meier survival curves of high-risk and low-risk groups in the validation cohorts. **(C)** Box plots showing immune populations in the CPAN-index groups. **(D)** Immune-score, Stromal-score and ESTIMATE-score in the CPAN-index groups. **(E)** Correlation between CPAN-index and immune cell infiltration in CRC. (*p < 0.05, **p < 0.01, ***p < 0.001, ****p < 0.0001).

GSN, CDKN2A, CAV1 and PDK4 were the risk factors of CRC, whereas the remaining CPAN_DEGs were protective factors (**Figure 6a**). Through LASSO regression, we selected 11 CPAN_DEGs, including CDKN2A, GZMB, PMAIP1, CASP5, ENDOD1, FAS, CASP7, CAV1, PDK4, CHP2, and PRKACB, to construct a prognostic model (**Figure 6b**). The coefficients of the CPAN-index genes in overall clinical outcome are shown in **Figure 6c**. The AUC of the model for predicting 1-, 3-, and 5-year survival of CRC patients in the training cohort were 0.62, 0.6, and 0.62 respectively, indicating good predictive performance (**Figure 6d**). The distribution of the CPAN index among the CRC patients, and the survival status of individual patients are shown in **Figure 6e**. Based on the expression patterns of the 11 model genes, the patients were stratified into the high-risk and low-risk groups (**Figure 6e**). The CPAN index showed exponential distribution across these three GEO validation sets, indicating a significant correlation between patient survival outcomes and the expression levels of the model genes (**Figure 7a**). Furthermore, patients classified as high-risk had significantly worse overall survival compared to the low-risk patients across all validation sets (p < 0.0001; **Figure 7b**), thereby confirming the robust predictive power of the CPAN-index model.

### 3.5 Immune profiling of CPAN-index risk groups

The risk groups also showed differences in immune infiltration. As shown in **Figure 7c**, the infiltration of CD4 memory resting T cells was notably diminished in the high-risk cohort compared to the low-risk group (p < 0.0001), M0 macrophages were more abundant in the high-risk samples relative to the low-risk samples (p < 0.001). However, no significant variations were observed in the immune scores, stromal scores, tumor purity, or ESTIMATE scores between the risk groups (**Figure 7d**). The CPAN index also correlated with multiple immune cell types, including follicular helper T cells, M1 macrophages, CD8 T cells, naïve B cells, regulatory T cells (Tregs), M2 macrophages, CD4 memory resting T cells, M0 macrophages, activated mast cells, resting NK cells, neutrophils, CD4 memory activated T cells, activated dendritic cells (DCs), CD4 naïve T cells, memory B cells, and eosinophils, gamma delta T cells. Furthermore, the monocytes, activated NK cells, plasma cells, resting DCs and resting mast cells were positively or negatively correlated with each other. We also observed a significant positive correlation between the T cell cluster and B cell cluster, specifically the memory B cells (p < 0.05), suggesting that these cells may work synergistically in mounting an immune response against CRC. In addition, M1 macrophages also showed a high positive correlation with M2 macrophages, which likely reflects their coordination in the inflammatory response (p < 0.05). In contrast, the relationship between NK cells and the T cell cluster was mostly negative (p < 0.05), suggesting antagonistic roles in immune response (**Figure 7e**). Taken together, the CPAN index is closely associated with the immune microenvironment of CRC.

**Figure 8:**
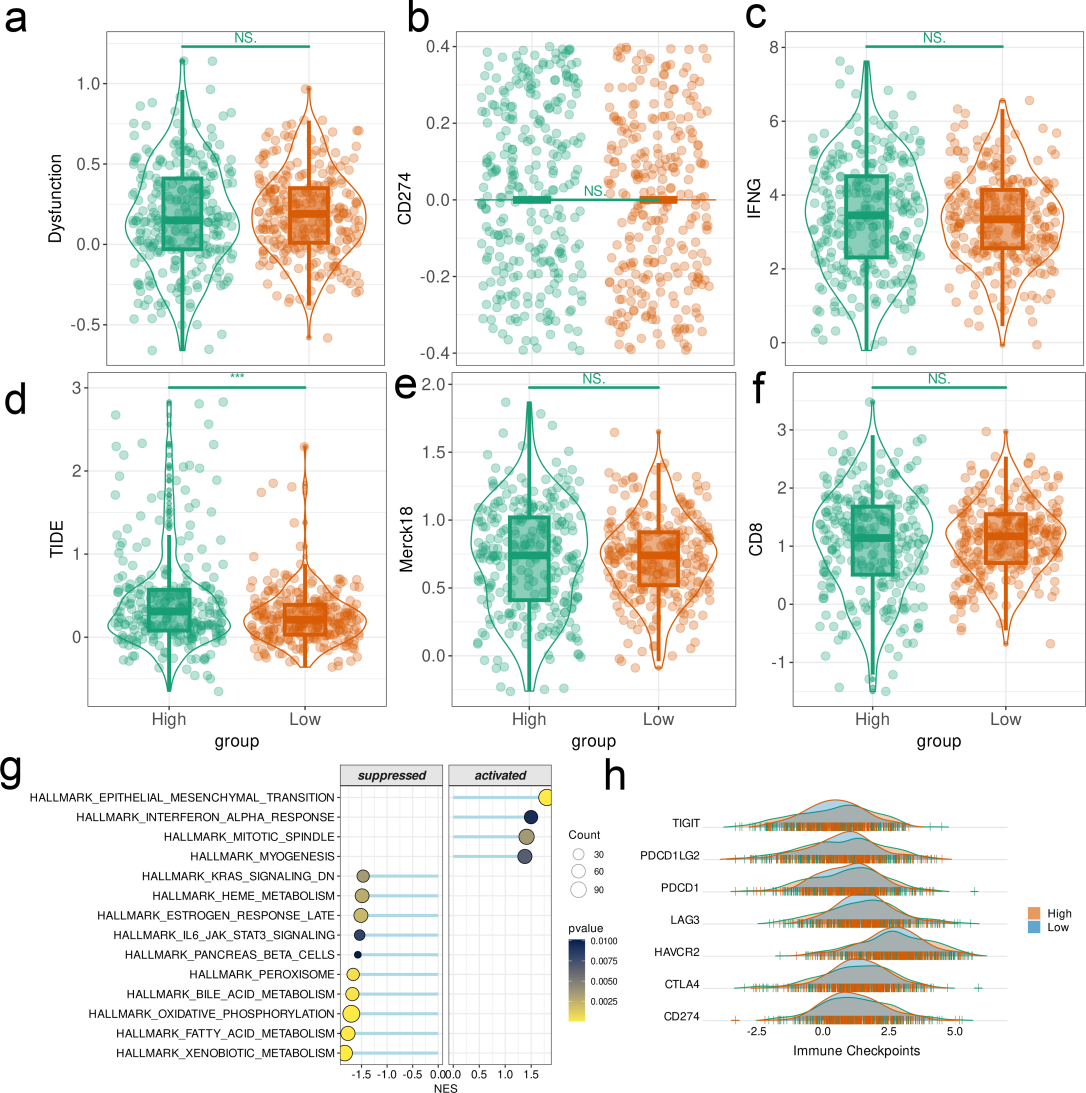
CPAN-index correlated with immune landscape and molecular heterogeneity of CRC. **(A–F)** TIDE, immune dysfunction, CD274, Merck18, CD8 and IFNγ scores in low-risk and high-risk groups. **(G)** Significantly enriched pathways in high-risk and low-risk groups. **(H)** Immune checkpoint expression in high-risk and low-risk groups. (*p < 0.05, **p < 0.01, ***p < 0.001).

The Dysfunction scores (**Figure 8a**), CD274 scores (**Figure 8b**), IFNG scores (**Figure 8c**), Merck18 scores (**Figure 8e**), and CD8 scores (**Figure 8f**) were similar in the two risk groups. Conversely, the TIDE score (**Figure 8d**) of the high-risk group was significantly higher than that of the low-risk group (p < 0.001), indicating a greater propensity of tumor immune escape in the former. Furthermore, the HALLMARK pathways related to the DEGs in the two risk groups were identified through GSEA (p < 0.0001, **Figure 8g**). The expression levels of TIGIT, PDCD1LG2, PD-L1, LAG3, HAVCR2, CTLA4 and CD274 were significantly different between the high-risk and low-risk samples (**Figure 8h**).

### 3.6 Single cell analysis

**Figure 9:**
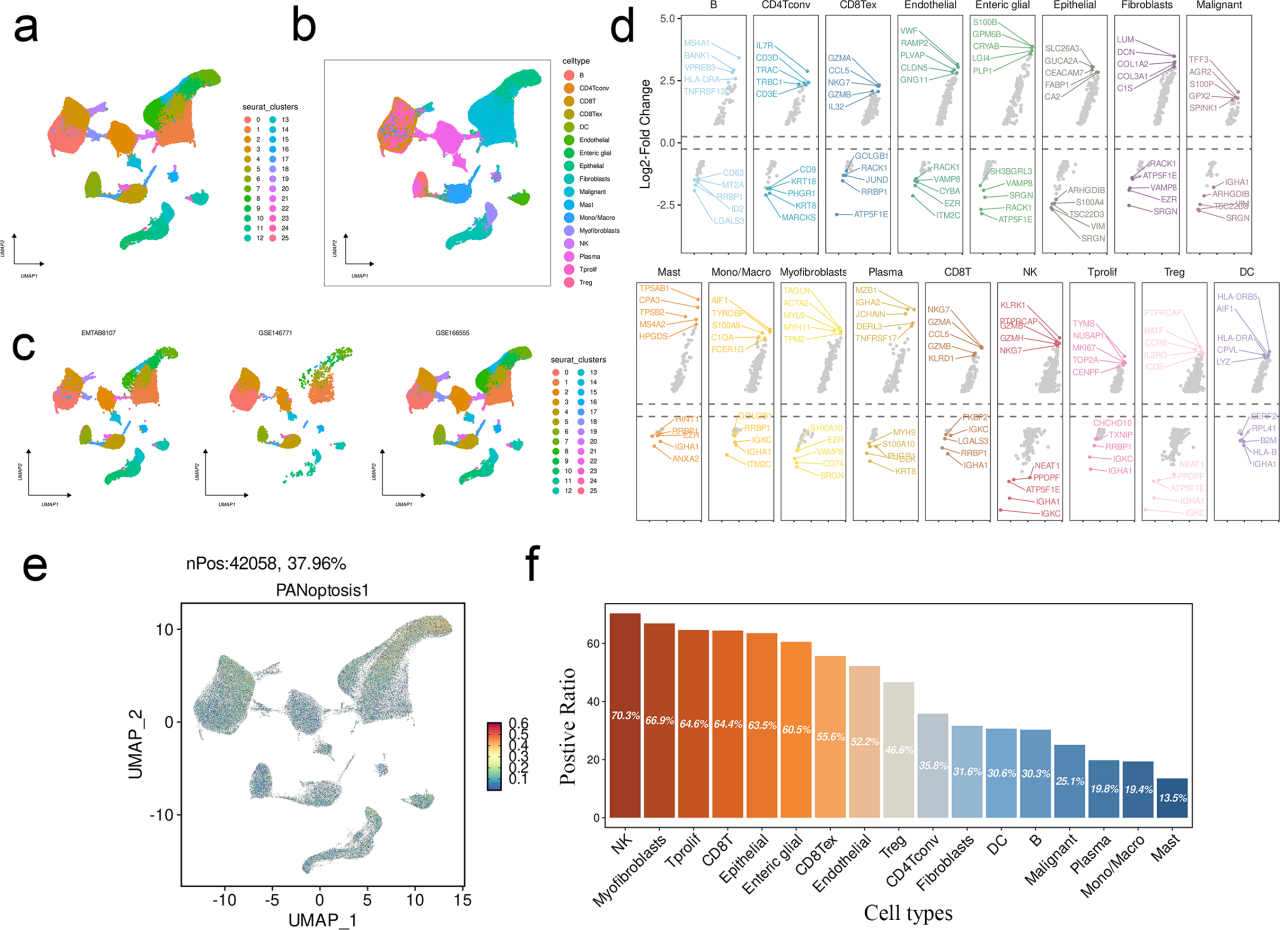
CPAN-index in single cell analysis. **(A–C)** UMAP visualization of cells from three common CRC scRNA-seq cohorts. **(D)** Volcanic maps showing downregulated and upregulated genes in each population. **(E)** Characteristic gene expression at the single-cell level measured by AddModuleScore () function in Seurat. **(F)** Histogram showing the proportion of CPAN-index positive cells in each population.

We performed dimensional-reduction clustering from three different scRNA-seq cohorts (EMTAB107, GSE146771, and GSE166555) using UMAP. The distribution of cell types was largely similar among the cohorts, indicating that there are common characteristics in the immune landscape of the CRC microenvironment. (**Figures 9a–c**). The up-regulated and down-regulated genes in each major cell type were also screened. MS4A1, BANK1, VPREB3, HLA-DRA, and TNFRSF13C were among the significantly up-regulated genes in B cells, whereas IL7R, CD3D, TRAC, TRBC1, CD3E, etc. showed obvious upregulation in the CD4+ T cells. In addition, CD9, KRT19, ANAX2, KLRD1, and RRBP1 were differentially expressed in various cell types (**Figure 9d**). The prognostic model in single cell datasets, and the score distribution in the different populations is shown in **Figure 9e**. We also calculated the proportion of cells positive for CPAN-index in each population. As shown in **Figure 9f**, NK cells had the highest positive rate of CPAN-index (70.3%), followed by myofibroblasts (66.9%), proliferative T cells (64.6%) and CD8 T cells (64.4%). In contrast, only 25.1% of the malignant cells and 13.5% of the mast cells were positive for CPAN-index (**Figure 9f**).

### 3.7 CDKN2A regulates the proliferation and migration of CRC cells

**Figure 10:**
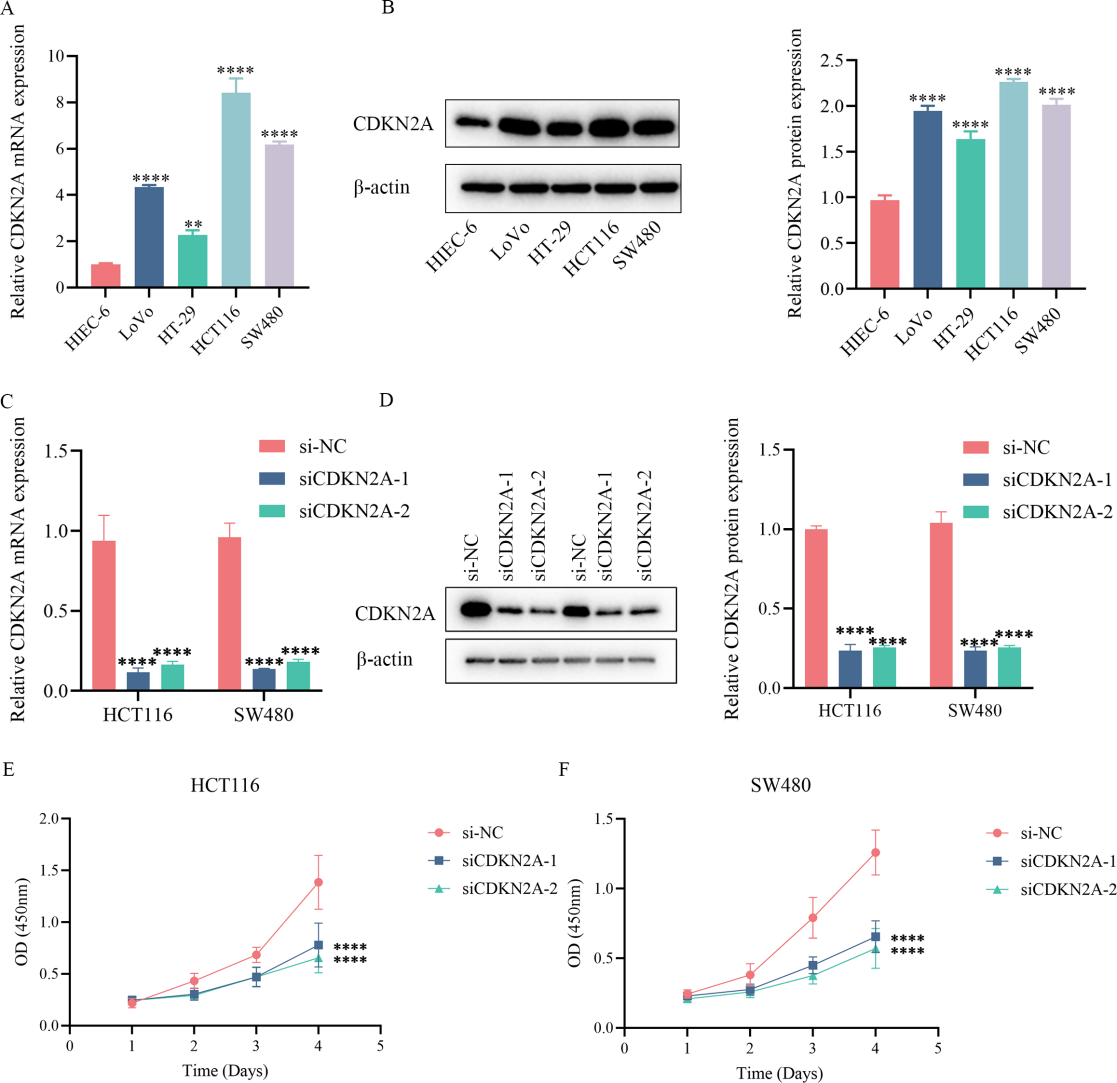
Expression and functional analysis of CDKN2A in colorectal cancer cell lines. **(A)** Relative expression levels of CDKN2A mRNA in the HIEC-6, LoVo, HT-29, HCT116 and SW480 cell lines. **(B)** Relative expression levels of CDKN2A protein in the HIEC-6, LoVo, HT-29, HCT116 and SW480 cell lines. **(C-D)** Relative CDKN2A mRNA and protein expression in the HCT116 and SW480 cells transfected with si-NC, siCDKN2A-1, or siCDKN2A-2 constructs. **(E)** Proliferation rates of HCT116 cells transfected with si-NC, siCDKN2A-1, or siCDKN2A-2 constructs. The line graph shows OD values at 450 nm over a period of 4 days. **(F)** Proliferation rates of SW480 cells transfected with si-NC, siCDKN2A-1, or siCDKN2A-2 constructs. The line graph shows OD values at 450 nm over a period of 4 days. ***p <0.01, ****p < 0.0001..

**Figure 11:**
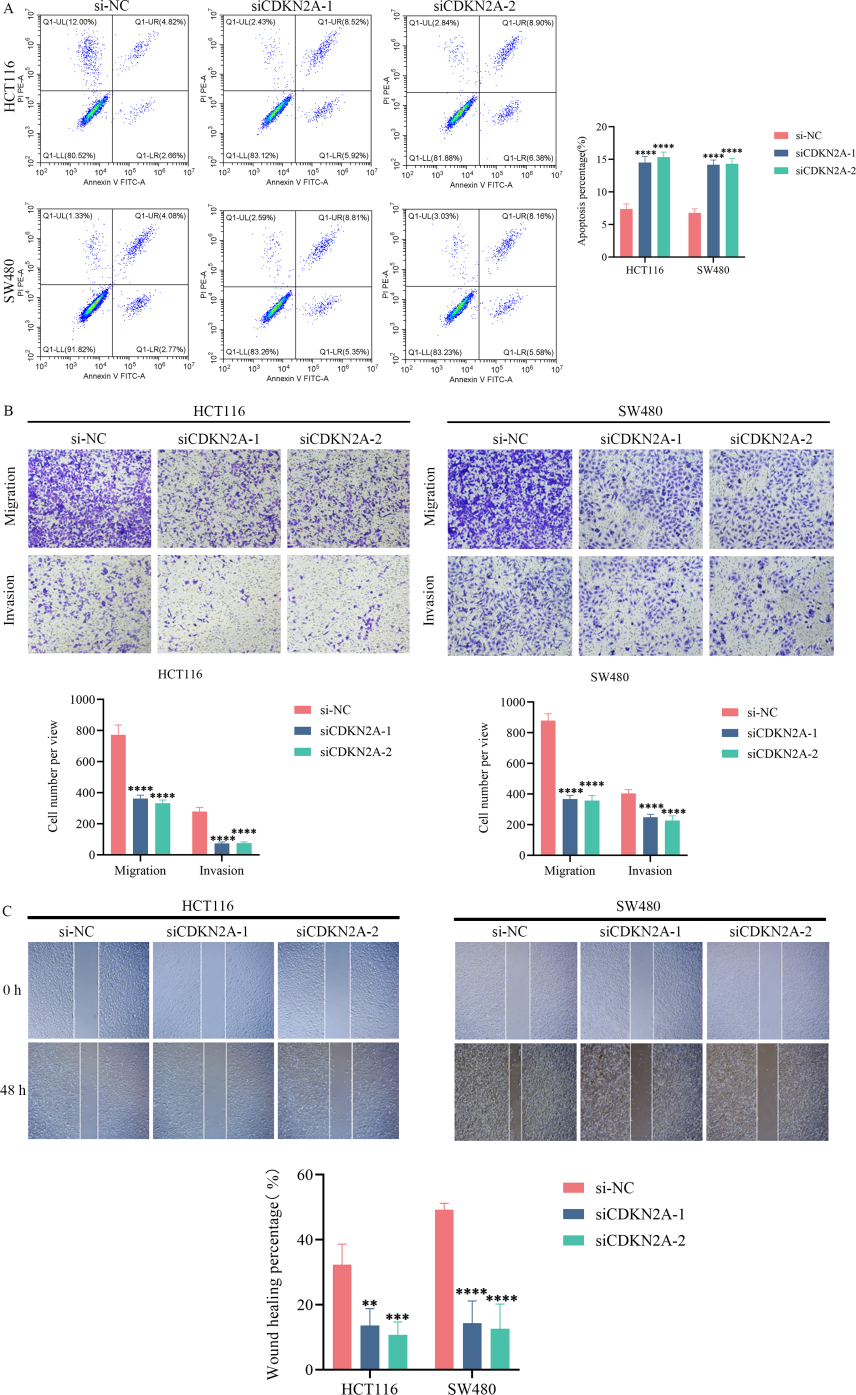
Impact of CDKN2A knockdown on apoptosis, invasion, and migration of colorectal cancer (CRC) cells. **(A)** Flow cytometry analysis showing the total apoptosis rate of CRC cells in two CDKN2A knockdown groups (transfected with siCDKN2A-1 and siCDKN2A-2) compared to the control group (transfected with si-NC). **(B)** Transwell assay results depicting the invasion and migration capabilities of CRC cells following CDKN2A knockdown. **(C)** Wound healing results demonstrating the effect of CDKN2A knockdown on CRC cell migration. *p < 0.05, **p < 0.01, ***p < 0.001, ****p < 0.0001.

Currently, research on the role of CDKN2A in CRC among model proteins is scarce, whereas the functions of other proteins have been well documented. This study focuses on CDKN2A as the research target to investigate its role in CRC. Firstly, we assessed its expression levels in four CRC cell lines and one normal colon epithelial cell line. The findings revealed that CDKN2A mRNA expression was notably higher in CRC cell lines compared to the control cell line (p < 0.01, **Figure 10A**). Western blot analysis further confirmed that CDKN2A protein expression was also significantly elevated relative to the control group (p < 0.0001, **Figure 10B**). Next, we constructed two si-CDKN2A nucleic acid systems to knock down CDKN2A expression in HCT116 and SW480 cell lines, laying the groundwork for subsequent experiments. We verified the effective knockdown efficiency of these two systems at both mRNA and protein levels through RTqPCR and Western blot assays (p < 0.0001, **Figures 10C, D**). CCK8 assay results indicated that after CDKN2A knockdown in the two CRC cell lines, absorbance decreased significantly, suggesting a marked inhibition of cell proliferation (p < 0.0001, **Figures 10E, F**). Flow cytometry demonstrated that the total apoptosis rate of cells in the two CDKN2A knockdown groups was significantly higher than that in the control group (p < 0.0001, **Figure 11A**). Transwell experiments confirmed a significant reduction in the invasion and migration capabilities of CRC cells following CDKN2A knockdown (p < 0.0001, **Figure 11B**). Scratch tests further corroborated the inhibitory effect of CDKN2A knockdown on cell migration (p < 0.01, **Figure 11C**).

## 4 Discussion

A greater understanding of the pathological mechanisms of CRC is essential to design better treatment strategies (30). In recent years, the role of cell death pathways in tumorigenesis and development has received significant attention. In this study, we analyzed the clinical significance of PANoptosis, a complex process involving apoptosis, pyroptosis, and necroptosis, in CRC, and constructed a prognostic model based the PANoptosis gene signature. The apoptosis-related genes comprised the major chunk of the PANoptosis gene set in CRC, indicating a significant role in cancer development and progression. In addition, the differentially expressed PANoptosis genes between the CRC and normal tissues (CPAN_DEGs) were significantly enriched in pathways related to cell death and responses to metabolic stress, as well as autoimmune diseases, thereby presenting novel targets for developing effective therapies.

Based on the expression levels of CPAN_DEGs, the CRC samples were stratified into two distinct clusters, which differed in terms of gene expression profiles and immune cell distribution, but showed similar survival rates and TMB. Thus, unsupervised clustering based on 15 CPAN_DEGs could not distinguish patient outcomes, which can be attributed to the complexity of the PANoptosis pathway, and the co-existence of pro-death and pro-survival signals. Using LASSO dimensionality reduction, we selected 11 core CPAN_DEGs, including CDKN2A, CASP5 and PDK4 among others, to construct a prognostic model. This CPAN-index model accurately stratified CRC patients into the high-risk and low-risk groups, indicating superior predictive efficacy. In addition, the prognostic model showed consistent performance across multiple independent datasets, indicating that its generalization ability and clinical translation potential. Therefore, the expression patterns of these PANoptosis gene markers can help identify high-risk CRC patients and guide individualized treatment strategies.

The high-risk group showed significantly higher TIDE score compared to the low-risk group, which is indicative of stronger immune escape potential due to T cell dysfunction or enhanced matrix barriers in the former (31, 32). Furthermore, the enrichment of resting NK cells and unpolarized macrophages in the high-risk samples may reflect an immunosuppressive state. Although the overall immune score of the microenvironment was not lower, the immune function was inhibited. On the other hand, the elevation of resting CD4 memory T cells and plasma cells in the low-risk samples could be indicative of potential future immune responses. Interestingly, the indicators of immunotherapy responses, such as CD8+ T cell levels and PD-L1 (CD274) expression, remained comparable between the two groups. This suggests that the prognostic significance of the CPAN-index may operate independently of established markers, and is likely related to the functional polarization of immune cells (including M0/M2 equilibrium and suppression of NK cell activity), as well as non-classical immune evasion pathways such as pyroptosis-driven chronic inflammation. In conclusion, CPAN-index can reveal differences in the immune landscape that cannot be captured by traditional microenvironment scores, and provide a new perspective for prognosis assessment and combination therapies targeting the immunometabolism-death interactive network.

The high-risk group showed significant heterogeneity in multiple HALLMARK pathways related to tumor progression and microenvironment remodeling. The activation of epithelial mesenchymal transformation (EMT) and mitotic spindle pathways suggests that the tumor cells in high-risk samples have stronger metastasis ability that may drive aggressive phenotypes through EMT-related transcription factors (such as SNAI1 and ZEB1), along with abnormal mitotic processes that may lead to increased genomic instability (27, 33). Upregulation of interferon alpha response is usually indicative of immune stress induced by chronic viral infection or endogenous retroelement activation. However, when considering the elevated TIDE scores and functional silencing of NK cells in high-risk samples, the interferon signaling pathway may be associated with immune tolerance or T cell depletion due to persistent overactivation rather than an effective anti-tumor immune response. It is worth noting that the enrichment of myogenic pathways in the high-risk samples may be due to the abnormal activation of cancer-associated fibroblasts, which promote stromal sclerosis and an immunosuppressive TME by secreting factors such as TGF-β. On the other hand, downregulation of the KRAS signaling inhibitory pathway suggested continuous activation of KRAS driving signals, which may promote cell proliferation and drug resistance through the MAPK/ERK pathway. The general inhibition of metabolic pathways is consistent with tumor cells relying on glycolysis (Warburg effect) and lipid synthesis reprogramming to meet their high energy demands. This metabolic pressure may exacerbate the acidity in the TME and inhibit immune cell function. In addition, the downregulation of IL6-JAK-STAT3 signaling in the high-risk samples was inconsistent with the enrichment of M2-type macrophages, which indicates that non-classical activation of STAT3, such as through the EGFR or inflammasome pathway, is the dominant driver of cancer. In addition, decreased metabolic capacity may impair the detoxification of chemotherapy drugs and lead to treatment resistance. Taken together, the molecular characteristics of the high-risk samples correlate with the transfer-metabolism-immunosuppression phenotype.

Single-cell analysis showed that a higher proportion of NK cells, CD8+ T cells and myofibroblasts were positive for the CPAN-index compared to tumor cells. This suggests that immune cells could not effectively kill tumors due to overactivation that eventually led to apoptosis or pyroptosis. In addition, the myofibroblasts likely contributed to the formation of a physical barrier and immunosuppressive TME by secreting pro-inflammatory and pro-fibrotic factors, and their metabolic abnormalities related to PANoptosis further aggravated chronic inflammation and tumor metastasis. At the same time, tumor cells escape immune-mediated clearance by downregulating programmed death genes, and the inflammation and metabolic stress in the TME provided an additional survival advantage. This vicious cycle of immune depletion, matrix remodeling, and tumor escape provides a new direction for targeted interventions, such as combining immune checkpoint inhibitors with anti-fibrotic drugs, or regulating PANoptosis.

The CDKN2A gene plays a complex and crucial role in the occurrence, progression, and treatment response of CRC (34). Its functional abnormalities drive malignant tumor phenotypes through multiple mechanisms, including cell cycle dysregulation, epigenetic modification, immune microenvironment remodeling, and metabolic reprogramming. CDKN2A exhibits a double-edged sword effect in CRC. Low expression leads to a loss of its tumor-suppressive function, promoting cell proliferation and invasion; high expression, however, may promote tumor progression through non-classical pathways. Mechanistic studies have found that high doses of CDKN2A can enhance tumor cell migration by activating the Wnt/β-catenin pathway or promoting goblet cell mucus secretion. Furthermore, CDKN2A is involved in immune escape in CRC. For example, the long non-coding RNA SNHG26 inhibits CD8+ T cell infiltration by degrading CDKN2A mRNA, while simultaneously promoting regulatory T cell (Treg) differentiation, creating an immunosuppressive microenvironment. This immunomodulatory effect is closely related to CDKN2A expression levels—high expression allows tumor cells to secrete IL-10, which can induce Treg differentiation, further weakening the anti-tumor immune response. Epigenetic modification is an important mechanism for the dysfunction of CDKN2A in CRC. Abnormal DNA methylation leads to CDKN2A silencing, while histone modification enhances the transcription of pro-inflammatory factors and promotes tumor-associated inflammation.

PANoptosis plays a crucial role in the tumor heterogeneity, immune remodeling, and prognosis in CRC, and the high-risk patients identified by the CPAN-index model present a metastatic-metabolic-immunosuppressive phenotype. The CPAN-index accurately captured the core network driving CRC progression by integrating the heterogeneity in PANoptosis genes, thus providing a theoretical basis for developing combination therapy strategies targeting the immunometabolism-death interaction network. In the future, it is necessary to further analyze the mechanism of action of key genes (such as CDKN2A) and promote clinical translational applications.

Our study offers valuable insights into how the CDKN2A mediated PANoptosis signaling network affects CRC progression, but has limitations. First, results mainly come from *in vitro* experiments with colon cancer cell lines; lack of *in vivo* validation limits extrapolation to living organisms’ complex physiological environments, as *in vivo* studies can consider factors like tissue architecture and tumor microenvironment interactions not replicated *in vitro*. Second, we haven’t directly measured PANoptosis and immune microenvironment changes after CDKN2A perturbation. Though our design and analysis imply their involvement, direct measurement methods would provide more definitive evidence and enhance understanding of CDKN2A perturbation’s precise mechanisms. Third, our study heavily relies on public datasets, which, while valuable, have inherent limitations like batch effects that may confound results and clinical heterogeneity among samples, introducing variability that could impact the generalizability and robustness of findings.

## 5 Conclusion

PANoptosis plays a key role in driving the malignant progression of CRC by regulating tumor heterogeneity, immune remodeling, and metabolic reprogramming. The CPAN-index model could stratify the patients based on their prognosis, and the high-risk patients were characterized by the upregulation of pathways related to cancer invasion, metabolic reprogramming, and immunosuppression. While this study provides a new direction for accurate prognosis assessment and targeted intervention strategies in CRC, the mechanisms of CDKN2A and other key genes need to be elucidated further to promote the clinical application of the CPAN-index model.

## Data Availability

The original contributions presented in the study are included in the article/supplementary material. Further inquiries can be directed to the corresponding author.
